# Enhancing indicator condition–guided HIV testing in Taiwan: a nationwide case–control study from 2009 to 2015

**DOI:** 10.1186/s12889-024-18499-6

**Published:** 2024-04-05

**Authors:** Chun-Yuan Lee, Yi-Pei Lin, Chun-Yu Lin, Po-Liang Lu, Fu-Wen Liang

**Affiliations:** 1 Division of Infectious Diseases, Department of Internal Medicine, Kaohsiung Medical University Hospital, Kaohsiung, Taiwan, R.O.C.; 2 School of Medicine, College of Medicine, Kaohsiung Medical University, Kaohsiung, Taiwan, R.O.C.; 3 M.Sc. Program in Tropical Medicine, College of Medicine, Kaohsiung Medical University, Kaohsiung, Taiwan, R.O.C.; 4 Graduate Institute of Medicine, College of Medicine, Kaohsiung Medical University, Kaohsiung, Taiwan, R.O.C.; 5 Center for Liquid Biopsy and Cohort Research, Kaohsiung Medical University, Kaohsiung, Taiwan, R.O.C.; 6 School of Post-Baccalaureate Medicine, College of Medicine, Kaohsiung Medical University, Kaohsiung, Taiwan, R.O.C.; 7 Department of Public Health, College of Health Science, Kaohsiung Medical University, Kaohsiung, Taiwan, R.O.C.; 8 Center for Big Data Research, Kaohsiung Medical University, Kaohsiung, Taiwan, R.O.C.; 9 Department of Medical Research, Kaohsiung Medical University Hospital, Kaohsiung, Taiwan, R.O.C.

**Keywords:** Acquired immunodeficiency syndrome (AIDS), Human immunodeficiency virus (HIV), Indicator condition, Late presentation, Testing

## Abstract

**Background:**

Although indicator condition (IC)-guided HIV testing (IC-HIVT) is effective at facilitating timely HIV diagnosis, research on IC categories and the related HIV risk in Taiwan is limited. To improve the adoption and spread of IC-HIVT in Taiwan, this study compared the IC categories of people living with HIV (PLWH) and non-HIV controls and investigated delays in the diagnosis of HIV infection.

**Methods:**

This nationwide, retrospective, 1:10-matched case–control study analyzed data from the Notifiable Diseases Surveillance System and National Health Insurance Research Database to evaluate 42 ICs for the 5-year period preceding a matched HIV diagnostic date from 2009 to 2015. The ICs were divided into category 1 ICs (AIDS-defining opportunistic illnesses [AOIs]), category 2 ICs (diseases associated with impaired immunity or malignancy but not AOIs), category 3 ICs (ICs associated with sexual behaviors), and category 4 ICs (mononucleosis or mononucleosis-like syndrome). Logistic regression was used to evaluate the HIV risk associated with each IC category (at the overall and annual levels) before the index date. Wilcoxon rank-sum test was performed to assess changes in diagnostic delays following an incident IC category by HIV transmission routes.

**Results:**

Fourteen thousand three hundred forty-seven PLWH were matched with 143,470 non-HIV controls. The prevalence results for all ICs and category 1–4 ICs were, respectively, 42.59%, 11.16%, 15.68%, 26.48%, and 0.97% among PLWH and 8.73%, 1.05%, 4.53%, 3.69%, and 0.02% among non-HIV controls (all *P* < 0.001). Each IC category posed a significantly higher risk of HIV infection overall and annually. The median (interquartile range) potential delay in HIV diagnosis was 15 (7–44), 324.5 (36–947), 234 (13–976), and 74 (33–476) days for category 1–4 ICs, respectively. Except for category 1 for men who have sex with men, these values remained stable across 2009–2015, regardless of the HIV transmission route.

**Conclusions:**

Given the ongoing HIV diagnostic delay, IC-HIVT should be upgraded and adapted to each IC category to enhance early HIV diagnosis.

**Supplementary Information:**

The online version contains supplementary material available at 10.1186/s12889-024-18499-6.

## Background

Human immunodeficiency virus (HIV) testing is the first step in the prevention and treatment of HIV infection [1]. To increase the likelihood of early HIV diagnosis, countries worldwide have implemented various testing strategies, including voluntary counseling and testing (VCT) [2], routine or opt-out testing (which is performed in all health-care settings except those in which the prevalence of undiagnosed HIV infection is less than 0.1%) [3, 4], and HIV testing guided by indicator conditions (ICs) [5, 6]. Although HIV testing is free and highly accessible in many countries, approximately 50% of people living with HIV (PLWH) in Asia–Pacific countries [7], including China [8] and Taiwan [9], and European countries [10] receive a delayed diagnosis.

Practically implementing the aforementioned testing strategies can be challenging. For example, the implementation of VCT may be hindered by barriers related to perceived transmission risk among sexually active individuals and their willingness to undergo HIV testing [9]. In addition, routing or opt-out testing may lead to increased testing rates [11] but may violate regulations regarding informed consent in several countries [12] and infringe upon the rights of individuals with HIV infection because of structural inequalities and stigmatization [13]. Therefore, an alternative testing strategy is required that can overcome these barriers to early HIV diagnosis. Because the majority of PLWH present to clinics with immunity-related impairments or high-risk behaviors before receiving their HIV diagnosis [14–16], in 2012, the HIV in Europe Conference in Copenhagen proposed that IC-guided HIV testing (IC-HIVT) be implemented [17]. In 2014, during the HIV in Europe Initiative, a set of guidelines were published regarding IC-HIVT for adults [18]. Although IC-HIVT is a cost-effective approach to identifying undiagnosed HIV [19–21], its implementation remains a major challenge in Australia [22], the United States [23, 24], and Europe [25–28].

In 1997, Taiwan established a nationwide anonymous VCT program to control the spread of HIV infection among sexually active populations [29]. Between 2005 and 2017, the Taiwanese government mandated HIV testing for specific populations with specific ICs. These populations included blood donors, military recruits, prisoners, pregnant women, individuals with newly diagnosed sexually transmitted diseases, people aged 15–49 years with newly diagnosed *Mycobacterium tuberculosis* infection, and patients with acute hepatitis. Although the percentage of patients with undiagnosed HIV infection in Taiwan decreased from 21.5% in 2012 to 12.1% in 2019 [30], the uniform trend observed in the late presentation of HIV during this period indicated that many individuals with HIV infection were unaware of their HIV status or were unwilling to undergo HIV testing [9, 31]. Therefore, current HIV testing strategies in Taiwan must be improved. IC-HIVT eliminates the need for obtaining a patient’s sexual history or conducting HIV risk assessments and therefore considerably reduces the barriers to HIV testing [5]. To increase the rate of adoption and achieve widespread use of IC-HIVT in Taiwan, the ICs [18] of the populations with and without HIV and HIV must be compared, and potential delays in the diagnosis of HIV infection must be addressed.

In this nationwide, retrospective case–control study, we compared PLWH and non-HIV controls in Taiwan in terms of the categories of ICs that they exhibited within 5 years before a matched HIV diagnosis date. Changes in diagnostic delays following an IC from a specific category were evaluated with consideration of HIV transmission routes. To identify missed opportunities for early HIV diagnosis, we examined the incidence of ICs from other categories before the incidence of ICs related to an acquired immunodeficiency syndrome (AIDS)-defining opportunistic illness (AOI).

## Methods

### Data sources

The Taiwan HIV/AIDS Database is a subset of the Notifiable Diseases Surveillance System (NDSS), which is a Taiwanese database maintained by the Taiwan Centers for Disease Control (TCDC). The NDSS is a national platform on which physicians can report communicable diseases, such as syphilis, gonorrhea, invasive pneumococcus, HIV infection, and AIDS. Physicians in Taiwan are required to register newly confirmed cases of HIV infection and AIDS within 24 h of diagnosis, with AIDS defined per the criteria established by the US Centers for Disease Control and Prevention in 1993 [32]. The Taiwan HIV/AIDS Database includes the sociodemographic data (e.g., date of birth, sex, home address, marital status, and occupation) and clinical data (e.g., HIV transmission route, HIV diagnosis date, and AIDS status) of patients. The current available data for HIV/AIDS cases in the Taiwan HIV/AIDS database is up to the year 2016.

Taiwan’s National Health Insurance program is a mandatory insurance program that has been providing insurance coverage to > 99% of Taiwan’s residents since 1995 [33], and the National Health Insurance Research Database (NHIRD) is a database that maintains data on NHI-related patient claims. These data are anonymized before they are released by the Health and Welfare Data Science Center, Ministry of Health and Welfare, Taiwan. With the approval of an institutional review board, researchers can apply to access the NDSS and NHIRD.

### Study design and setting

The present case–control study analyzed patients who received a new diagnosis of HIV between January 1, 2009, and December 31, 2015. Patients eligible for inclusion as cases in the present study were selected from the HIV/AIDS Database. We excluded individuals aged < 15 years or ≥ 80 years, those who had already left Taiwan, and those with an unknown or a blood-transfusion HIV transmission route. The date of HIV diagnosis was defined as the index date. The control group comprised individuals who were identified from the NHIRD and did not have HIV, as indicated by the absence of the diagnostic code for HIV in their profiles in the Taiwan HIV/AIDS Database. To reduce selection bias and improve the statistical power for detecting differences in rare IC events [34], we matched the patients (cases) to control individuals at a 1:10 ratio by performing propensity score matching on the basis of age, sex, and index date (month/year). The propensity score was calculated using multivariable logistic regression to create the probability of being a cases and a greedy nearest neighbor matching without replacement was used to identify the closest available matches via SAS matching macro, “%OneToManyMTCH” [35]. Finally, we investigated the prevalence or incidence of ICs in the two groups within the 5-year period preceding the matched index date. This 5-year period was selected on the basis of the results of another study [36]. To evaluate the effects of AOI-related ICs on the all-cause mortality rate of the included patients, we analyzed the case group’s data for the period from the date of HIV diagnosis to patient death or December 31, 2016, whichever occurred first.

### Variable definitions and data collection

From the NHIRD, we retrieved data on four baseline comorbidities, namely cerebral vascular disease, chronic obstructive pulmonary disease, diabetes mellitus, and renal disease. In the present study, a patient was regarded as having a comorbidity if the condition occurred in an inpatient setting or was noted in ≥ 3 outpatient visits [37]. The IC data retrieved from the NHIRD and NDSS were screened using the list published by the European Centre for Disease Prevention and Control [18]. Given the higher seroprevalence of *Entamoeba histolytica* in PLWH than in non-HIV individuals [38] and the increased risk of shigellosis among PLWH and men who have sex with men (MSM) [39], we included *E. histolytica* and *Shigella* infections as ICs in the present study. Because several ICs were challenging to identify using the diagnostic codes of the *International Classification of Diseases*, *Ninth Revision*, or the NDSS, we preselected 42 ICs from the NHIRD and NDSS (Additional file 1). A patient was regarded as having an IC if they had ≥ 1 inpatient claims record or ≥ 3 outpatient claims records in the NHIRD that had IC codes that matched those of the *International Classification of Diseases*, *Ninth Revision* or any record of confirmed ICs in the NDSS. The date of IC incidence, as recorded in the NHIRD, or the date of a confirmed IC diagnosis, as recorded in the NDSS, was regarded as the IC date. According to NHIRD regulations, the results obtained from < 3 patients cannot be exported to prevent identification [40]. Thus, for ICs that were only noted in < 3 patients in the control or case group, the related data were combined with data classified under other categories (Additional file 1). The 42 preselected ICs were divided into four categories on the basis of related public health interventions, namely category 1 (AOIs), category 2 (diseases associated with impaired immunity or malignancy but not with AOIs), category 3 (ICs associated with sexual behaviors), and category 4 (mononucleosis or mononucleosis-like syndrome). The case group was stratified by HIV transmission route into the subgroups of MSM, heterosexuals, and people who inject drugs.

### Primary and secondary outcomes

The primary outcomes were the intergroup differences in IC categories and the associations of HIV infection with each IC category (overall association and association during each of the 5 years before the index date) for the period from 2009 to 2015. The secondary outcomes were the changes in potential delay in HIV diagnosis following the incidence of an IC from an IC category among PLWH with consideration of the route of HIV transmission. Finally, because the incidence of AOI-related ICs indicates advanced HIV infection, characterized by severe immunodeficiency [41], we focused on evaluating the effects of AOI-related ICs on the rate of all-cause mortality among PLWH as well as on analyzing the development of other categories of ICs before the incidence of AOI-related ICs.

### Statistical analysis

The results for categorical variables were presented as frequency and percentage values and were compared between groups by performing χ^2^ tests or Fisher’s exact tests. Binary logistic regression was performed to identify the associations of each IC and IC category with HIV infection [42] and to estimate the related crude and adjusted odds ratios with 95% confidence intervals (CIs), thereby allowing for the effect of each variable to be evaluated in terms of magnitude and direction. The changes in diagnostic delay following the incidence of an IC for the 2009–2015 period were analyzed using the Wilcoxon rank-sum test. The effects of AOI-related ICs on the all-cause mortality rate of the included patients were analyzed by employing Cox proportional-hazards models to evaluate the obtained crude and adjusted hazard ratios (HRs) with 95% CIs. All tests were two-tailed. A *P*-value of < 0.05 was regarded as significant. All data analyses were managed and performed using SAS (version 9.4; SAS Institute, Cary, NC, USA).

## Results

### Cohort formation

**Fig. 1:**
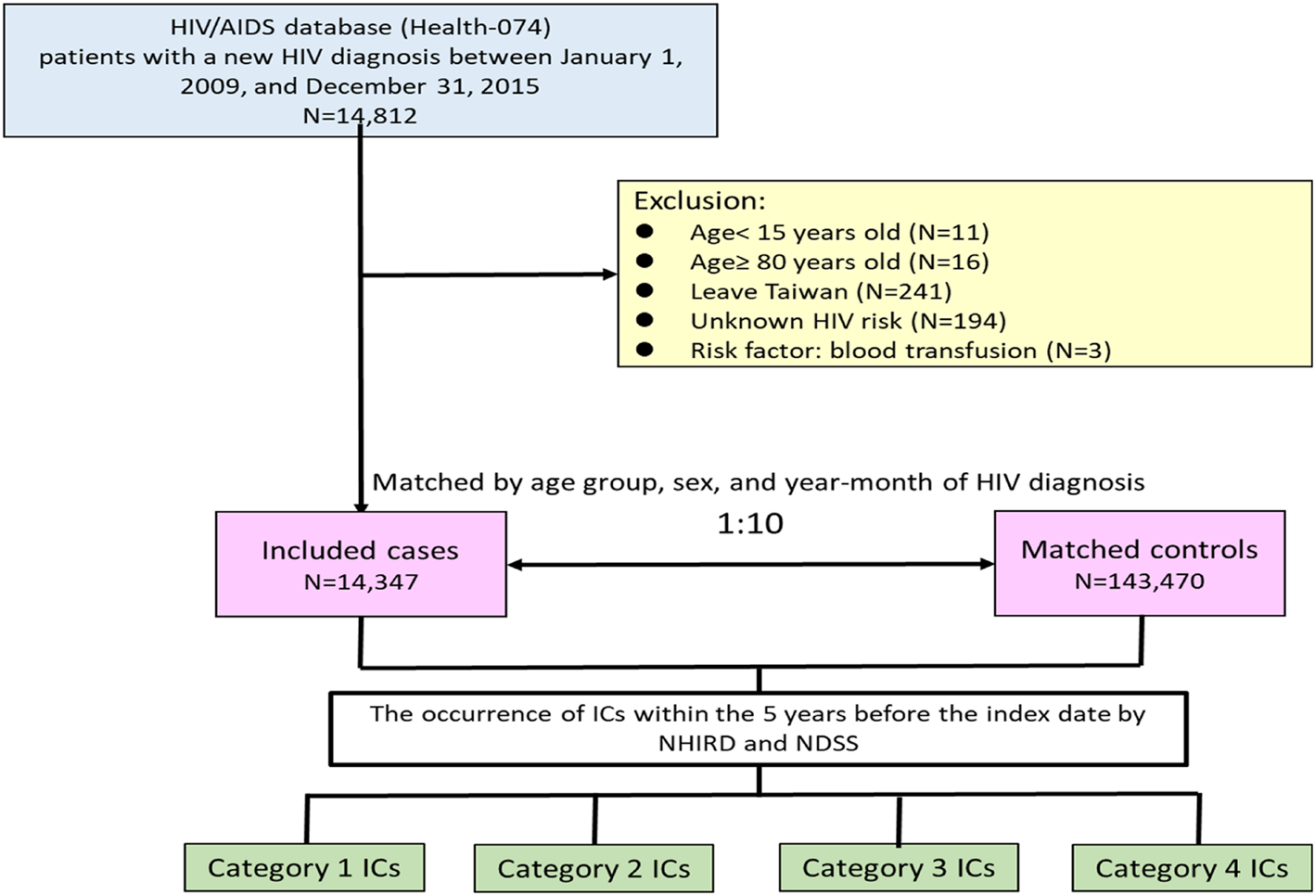
Study flowchart. Abbreviation: AIDS, acquired immunodeficiency syndrome; HIV, human immunodeficiency virus; NDSS, Notifiable Diseases Surveillance System; NHIRD, National Health Insurance Research Database

In total, 14,812 patients with newly diagnosed HIV were identified from the Taiwan HIV/AIDS Database. Among them, 465 were excluded for the following reasons: 11 were aged under 15 years, 16 were aged ≥ 80 years, 241 had already left Taiwan, 194 had unknown routes of HIV transmission, and 3 developed HIV infection through blood transfusion. Finally, 14,347 patients were included and matched with 143,470 non-HIV controls (Fig. 1).

### Cohort characteristics

In the case group, 96.56% were men, and 42.49% were aged 25–35 years. The prevalence rates for cerebral vascular disease, diabetes mellitus, and impaired renal function at baseline were higher in the control group than in the case group (all *P* < 0.001; Table 1).

**Table 1:** Comparison of sociodemographic characteristics of patients in case and control groups

	All (*n* = 157,817)	Control group (*n* = 143,470)	Case group (*n* = 14,347)	*P*-value
Age group (years old)				0.999
15≦ ~ < 25	46,530 (29.48)	42,300 (29.48)	4,230 (29.48)	
25≦ ~ < 35	67,056 (42.49)	60,960 (42.49)	6,096 (42.49)	
35≦ ~ < 45	28,787 (18.24)	26,170 (18.24)	2,617 (18.24)	
45≦ ~ < 55	10,967 (6.95)	9,970 (6.95)	997 (6.95)	
> 55	4,477 (2.84)	4,070 (2.84)	407 (2.84)	
Male gender	152,383 (96.56)	138,530 (96.56)	13,853 (96.56)	> 0.999
Calendar year of the index date				> 0.999
2009	18,028 (11.42)	16,250 (11.43)	1,625 (11.33)	
2010	19,708 (12.49)	17,920 (12.49)	1,792 (12.49)	
2011	21,577 (13.67)	19,580 (13.67)	1,958 (13.65)	
2012	24,216 (15.34)	21,990 (15.35)	2,199 (15.33)	
2013	24,479 (15.51)	22,260 (15.51)	2,226 (15.52)	
2014	24,610 (15.59)	22,330 (15.60)	2,233 (15.56)	
2015	25,199 (15.97)	23,140 (15.95)	2,314 (16.13)	
Comorbidities				
Cerebral vascular disease	1,841 (1.17)	1,730 (1.21)	111 (0.77)	< 0.001
Chronic lung disease	7,963 (5.05)	7,237 (5.04)	726 (5.06)	0.933
Diabetes mellitus	6,375 (4.04)	6,052 (4.22)	323 (2.25)	< 0.001
Renal disease	1,389 (0.88)	1,320 (0.92)	69 (0.48)	< 0.004

### Comparison of ICs and overall association of HIV infection with each IC

The prevalence rates for all selected ICs, with the exception of mononeuritis, peripheral neuropathy, oral hairy leukoplakia, other_category2_, hepatitis A virus infection, and hepatitis B virus infection, were higher in the PLWH than in the non-HIV controls. The most common ICs in categories 1, 2, and 3 were *Pneumocystis jirovecii* pneumonia (5.88%), herpes zoster infection (5.84%), and syphilis (19.75%), respectively.

**Fig. 2:**
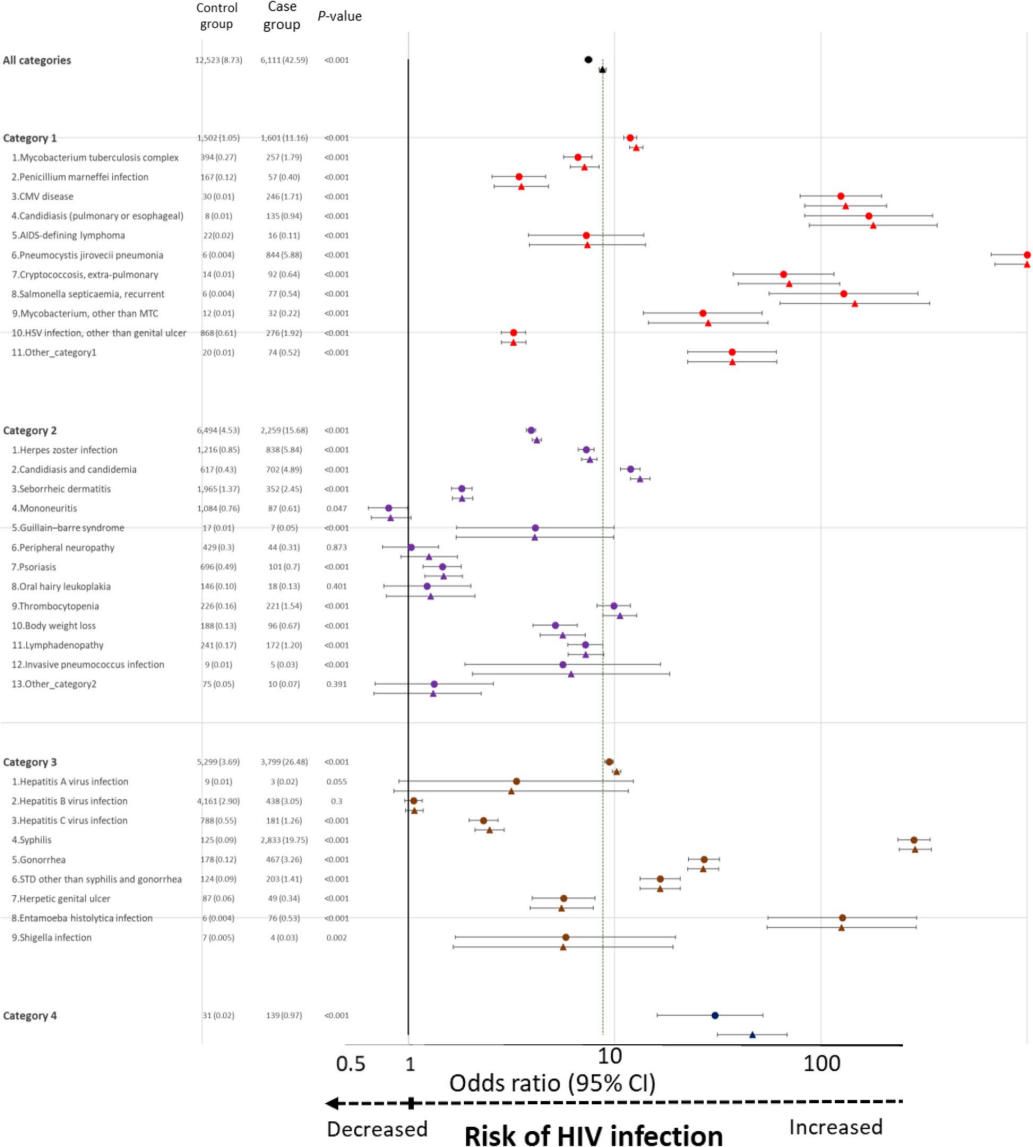
Association between HIV diagnosis and prevalence of each IC within 5 years before index date. Note: A circle (●) indicates a crude odds ratio; a triangle (▲) indicates an adjusted odds ratio. Abbreviations: HIV, human immunodeficiency virus; CI, confidence interval; CMV, cytomegalovirus; HSV, herpes simplex virus; IC, indicator condition; MTC,* Mycobacterium tuberculosis* complex; STD, sexually transmitted disease

All selected ICs, with the exception of mononeuritis, peripheral neuropathy, oral hairy leukoplakia, other_category2_, hepatitis A virus infection, and hepatitis B virus infection, were associated with an increased risk of HIV infection (Fig. 2) (Additional file 2).

### Comparison of each IC category and the association of HIV infection with each IC category overall and throughout the five consecutive 1-year intervals before the index date

**Fig. 3:**
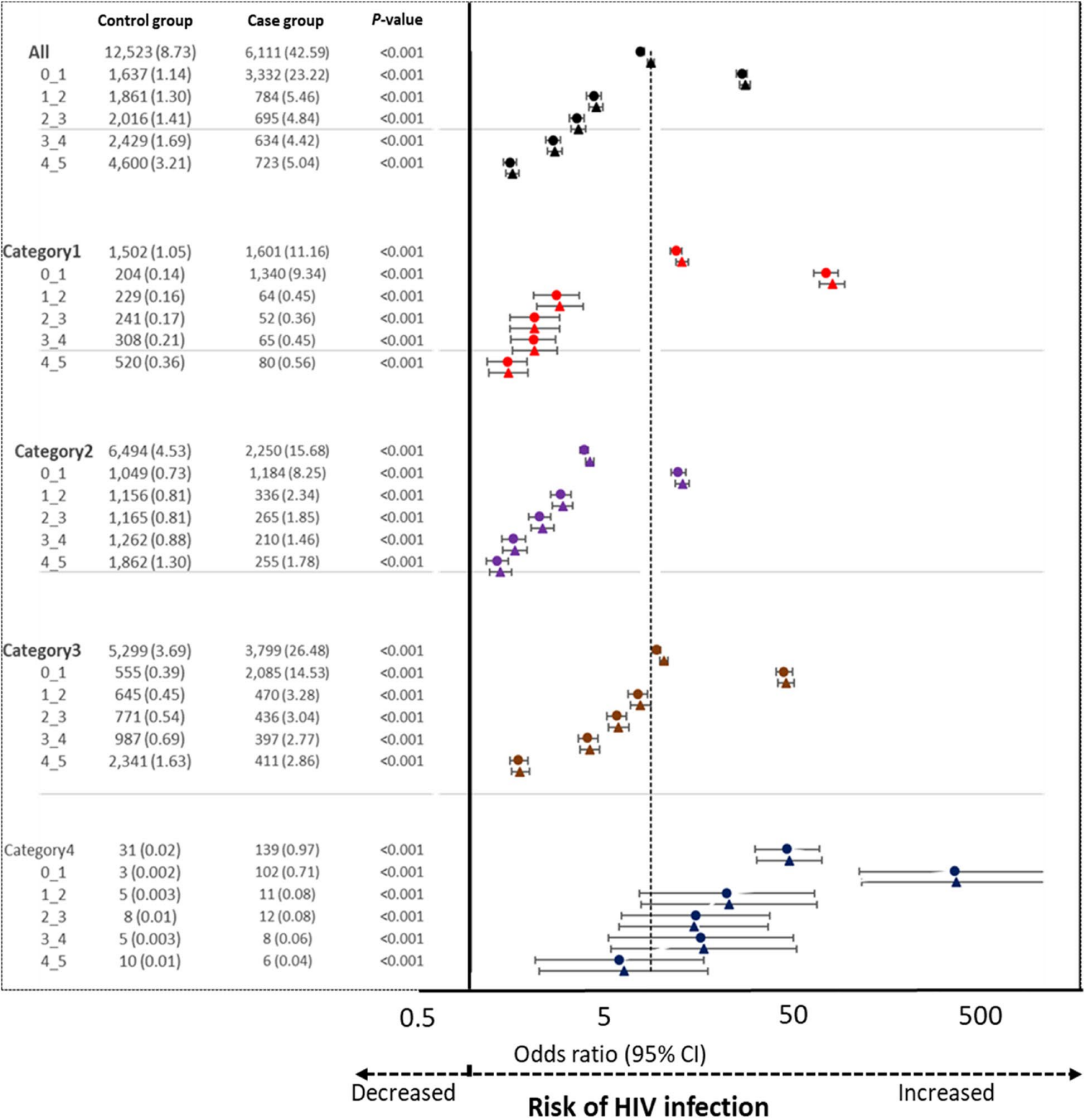
Association between HIV diagnosis and prevalence of ICs from each IC category across 5 consecutive 1-year intervals from index date. Note: A circle (●) indicates a crude odds ratio; a triangle (▲) indicates an adjusted odds ratio. Abbreviations: CI, confidence interval; HIV, human immunodeficiency virus

Overall, 42.59% of the PLWH developed at least one IC, whereas only 8.73% of the non-HIV controls experienced the same IC (*P* < 0.001). Furthermore, in terms of the percentage of patients who developed at least one IC, the results are as follows. Among the PLWH, 11.16%, 15.68%, 26.48%, and 0.97% developed at least one IC from category 1, 2, 3, and 4, respectively; among the non-HIV controls, 1.05%, 4.53%, 3.69%, and 0.02% developed at least one IC from category 1, 2, 3, and 4, respectively (all *P* < 0.001). Overall, all IC categories were associated with an increased risk of HIV infection (Fig. 3) (Additional file 3).

The annual prevalence of ICs from each IC category during the 5-year period was significantly higher in the PLWH than in the non-HIV controls. The positive association between the annual prevalence of ICs from each IC category and the risk of HIV infection persisted throughout the 5-year period.

### Changes in potential delay in HIV diagnosis following incidence of IC from an IC category (overall results and results stratified by HIV transmission route)

The median (interquartile range [IQR]) delay period was 265 days (16–989 days) for all ICs, 15 days (7–44 days) for category 1 ICs, 324.5 days (36–947 days) for category 2 ICs, 234 days (13–976 days) for category 3 ICs, and 74 days (33–476 days) for category 4 ICs. From 2009 to 2015, the trend for the delay in HIV diagnosis remained stable for all IC categories, with the exception of category 2 ICs (*P* = 0.042; Table 2).

**Table 2:** Change in HIV diagnostic delay following incidence of IC from each IC category among PLWH (results stratified by year and HIV transmission routes)

	Overall	2009	2010	2011	2012	2013	2014	2015	*P*-value
All routes of HIV transmission, median day (IQR)									
Category 1 IC	15 (7–44)	15.5 (7.5–115.5)	18 (8–42)	13 (6–30)	15 (7–103)	16 (7–43)	14 (7–28)	14 (8–43.5)	0.294
Category 2 IC	324.5 (36–947)	341 (30–864)	369 (47–1,042)	197 (23–778)	304 (46–980)	325 (37–887)	376 (38–940)	343.5 (33–1,163)	0.042
Category 3 IC	234 (13–976)	300 (14–1,074)	229 (12–1,076)	216 (14–909)	256 (12–928)	218.5 (14–906)	310 (13–1,020)	145 (12–1,011.5)	0.533
Category 4 IC	74 (33–476)	81 (34.5–817.5)	176.5 (69–623)	96.5 (34–357)	90 (31–268)	46 (22–140)	68 (41–227)	44 (19–1,296)	0.605
All ICs	265 (16–989)	334.5 (18–1,046)	281 (17–1,064)	198 (16–870)	266 (16–988.5)	277 (18–949)	323 (17–989)	209 (14–1058)	0.193
MSM, median day (IQR)									
Category 1 IC	15 (7–37)	17 (8–530)	16.5 (8–30)	11.5 (5–25.5)	15 (7–62)	16 (8–65)	13 (7–21)	14 (8–44)	0.043
Category 2 IC	278 (33–860)	219 (23–771)	248 (40–902)	192.5 (18–712.5)	242 (46–904)	325 (38–825)	346 (37–896.5)	333 (29–1,107)	0.105
Category 3 IC	213 (13–953)	245 (13–1,077)	184 (12–1,012)	212 (15–909)	249 (12–910)	208.5 (14–881)	301 (13–1,019)	138 (12–857)	0.620
Category 4 IC	74 (32–370)	72 (33–970)	188 (32–839)	96.5 (24–304.5)	90 (39–217)	46 (18–224)	67.5 (27–199.5)	50.5 (17.5–1,304)	0.700
All ICs	241 (16–956)	265 (17–1,052)	210 (16–996)	198 (15–838)	248 (16–944)	262 (18–917)	308 (16–958)	203 (14–1,032)	0.480
PWID, median day (IQR)									
Category 1 IC	29 (8–435.5)	39 (5.5–272.5)	88 (3–93)	89 (14–681)	10 (9–709)	879.5 (16–1,743)	25.5 (21–41)	23.5 (6–605)	0.961
Category 2 IC	696 (205–1,243)	468.5 (302–1,025)	884 (634.5–1,307)	263 (168.5–609.5)	387 (224–1,143)	1,028.5 (780–1,286.5)	1,596 (717.5–1,776.5)	689 (118–1,277)	0.251
Category 3 IC	589 (101–1,214)	727 (101–1,083)	872.5 (233–1,334)	417.5 (9–727)	907.5 (476–1,549)	357 (17–947)	519 (110–884)	816.5 (126–1,577)	0.326
Category 4 IC	1,165 (1,165–1,165)			1,165 (1,165 –1,165 )					N/A
All ICs	620.5 (108–1,246.5)	581 (157–1,083)	823 (135–1,336)	410 (97–941)	1,079 (443–1,508)	780 (20–1,238)	486.5 (41–1,108)	760 (108–1,490)	0.431
Heterosexuals, median day (IQR)									
Category 1 IC	16.5 (7–52)	15 (7–37)	20.5 (8–62)	19 (7–32)	19.5 (6–230)	15 (6–25)	16 (7–270)	14 (9–31.5)	0.837
Category 2 IC	417.5 (38.5–1,142)	423 (81.5–1,037)	569 (77–1,154.5)	182 (23–972)	495 (29–1,262)	293.5 (32–1,011)	561 (64–1,175)	421 (33–1,216)	0.365
Category 3 IC	250.5 (11–1,095)	339 (11–1,011)	428.5 (11–1,279)	210 (12–831)	230 (12–921)	288 (10–1,164)	347.5 (11–1,098)	137 (10–1,168)	0.990
Category 4 IC	76 (40.5–543)	567 (567–567)	165 (88–204)	64 (48–865)	271.5 (24–519)	41.5 (22–61)	648 (648–648)	33.5 (33–34)	0.302
All ICs	324 (18–1,141)	337 (22–1,012)	391.5 (21–1,208)	155 (19–951)	368 (19–1,149)	279 (19–1,156)	514 (16–1,175)	243 (13–1,216)	0.676

According to NHIRD regulations, the results obtained from < 3 patients cannot be exported to prevent identification [40]. As a result, blank cells indicate “fewer than 3 patients”

*Abbreviations: IC* indicator condition, *IQR* interquartile range, *MSM* men who have sex with men, *N/A* not available, *PLWH* people living with HIV, *PWID* people who inject drugs

A subgroup analysis revealed that the delay in HIV diagnosis among the patients remained consistent between 2009 and 2015 for all IC categories, except for category 1 for the MSM, regardless of the route of HIV transmission (Table 2). Among the MSM, the median (IQR) delay in HIV diagnosis following the incidence of a category 1 IC decreased slightly from 17 (8–530) days in 2009 to 14 (8–44) days in 2015 (*P* = 0.043).

### Effects of category 1 ICs on all-cause mortality rate among PLWH

The prevalence of category 1 ICs among the PLWH was significantly associated with an increased risk of all-cause mortality in both the crude model (crude HR, 4.44; 95% CI, 3.88–5.09; *P* < 0.001) and the adjusted model (adjusted HR, 3.08; 95% CI, 2.65–3.57; *P* < 0.001; Table 3).

**Table 3:** Effects of category 1 ICs on all-cause mortality rate among PLWH

	Crude HR (95% CI)	*P*-value	Adjusted HR^a^ (95% CI)	*P*-value
Age group				
15≦ ~ < 25	Reference		Reference	
25≦ ~ < 35	2.20 (1.74–2.79)	< 0.001	1.62 (1.25–2.11)	< 0.001
35≦ ~ < 45	4.96 (3.91–6.29)	< 0.001	2.78 (2.11–3.67)	< 0.001
45≦ ~ < 55	8.77 (6.80–11.30)	< 0.001	3.69 (2.72–5.01)	< 0.001
> 55	18.43 (14.12–24.05)	< 0.001	5.58 (3.95–7.89)	< 0.001
Male gender (vs. female gender)	0.48 (0.37–0.62)	< 0.001	1.12 (0.85–1.47)	0.416
Cerebral vascular disease (vs. no)	5.81 (4.13–8.19)	< 0.001	1.50 (1.03–2.18)	0.033
Chronic lung disease (vs. no)	1.94 (1.55–2.43)	< 0.001	1.01 (0.80–1.28)	0.905
Diabetes mellitus (vs. no)	4.96 (3.97–6.20)	< 0.001	1.68 (1.32–2.15)	< 0.001
Renal disease (vs. no)	6.87 (4.65–10.15)	< 0.001	1.69 (1.10–2.60)	0.017
HIV transmission routes				
PWID	Reference		Reference	Reference
MSM	0.21 (0.18–0.25)	< 0.001	0.40 (0.31–0.50)	< 0.001
Heterosexual contact	0.67 (0.55–0.81)	< 0.001	0.54 (0.42–0.68)	< 0.001
Calendar year of the index date				
2009	Reference		Reference	
2010	0.83 (0.67–1.02)	0.071	0.90 (0.73–1.11)	0.334
2011	0.86 (0.69–1.06)	0.149	0.98 (0.79–1.21)	0.858
2012	0.72 (0.58–0.91)	0.005	0.86 (0.68–1.07)	0.178
2013	0.62 (0.49–0.79)	< 0.001	0.79 (0.62–1.01)	0.058
2014	0.62 (0.48–0.81)	< 0.001	0.79 (0.61–1.03)	0.08
2015	0.48 (0.36–0.65)	< 0.001	0.58 (0.43–0.78)	< 0.001
Marriage				
No	Reference		Reference	Reference
Unknown	0.52 (0.07–3.66)	0.509	0.41 (0.06–2.96)	0.379
Yes	3.92 (3.42–4.49)	< 0.001	1.09 (0.90–1.31)	0.392
Employment				
No	Reference		Reference	
Student	0.15 (0.10–0.21)	< 0.001	0.59 (0.39–0.88)	0.011
Unknown	0.75 (0.60–0.93)	0.009	0.97 (0.78–1.22)	0.809
Yes	0.51 (0.44–0.58)	< 0.001	0.79 (0.67–0.93)	0.005
Specimen source				
HIV referral center	Reference		Reference	
Military screening	0.22 (0.12–0.43)	< 0.001	0.58 (0.29–1.14)	0.047
Blood donation center	0.30 (0.16–0.55)	< 0.001	0.49 (0.26–0.91)	0.024
Jail screening	1.90 (1.48–2.45)	< 0.001	0.58 (0.29–1.14)	0.113
Others			1.19 (1.00–1.41)	0.047
HIV diagnosis region				
Kaoping area	Reference		Reference	Reference
Central Taiwan	1.07 (0.87–1.31)	0.515	1.13 (0.92–1.40)	0.235
Eastern Taiwan	1.30 (0.83–2.02)	0.251	1.42 (0.91–2.23)	0.120
Northern Taiwan	0.92 (0.74–1.15)	0.476	1.05 (0.84–1.32)	0.662
Southern Taiwan	1.10 (0.86–1.41)	0.444	0.95 (0.74–1.21)	0.665
Taipei area	0.73 (0.61–0.88)	< 0.001	0.86 (0.72–1.04)	0.115
Category 1 ICs (vs. no)	4.44 (3.88–5.09)	< 0.001	3.08 (2.65–3.57)	< 0.001

*Abbreviations: CI* confidence interval, *HIV* human immunodeficiency virus, *HR* hazard ratio, *IC* indicator condition, *MSM* men who have sex with men, *PLWH* people living with HIV, *PWID* people who inject drugs

^a^The Cox proportional-hazards model was adjusted for age, year of HIV diagnosis, sex, at-risk population, marriage, occupation, specimen source, region of HIV diagnosis, comorbidities, and category 1 IC

### Prevalence of category 2 and 3 ICs before incidence of category 1 ICs

Of the 1,601 PLWH with category 1 ICs, 641 (40.4%) developed category 2 ICs before the incidence of category 1 ICs (median number of days between the incidence of the two ICs [IQR], 58 [0–651] days), and 262 (16.4%) developed category 3 ICs before the incidence of category 1 ICs (median number of days between the incidence of the two ICs [IQR], 403 [4–1,130] days; Additional file 4).

## Discussion

Although a set of guidelines on IC-HIVT for adults were published during the HIV in Europe Initiative in 2014 [18], the implementation rate for IC-HIVT in Western countries remains low [43, 44]. In this study, we discovered that the potential delay in HIV diagnosis was 15 days (7–44 days) for category 1 ICs, 324.5 days (36–947 days) for category 2 ICs, and 74 days (33–476 days) for category 4 ICs. With the exception of AOI-related ICs among MSM, the potential delay in HIV diagnosis remained stable from 2009 to 2015 for category 1, 2, and 4 ICs, regardless of the transmission route of HIV. This finding indicates that even with the adoption of mandatory HIV testing for specific populations with specific ICs, the effectiveness of IC-HIVT for populations who are at a risk of HIV infection in Taiwan remains low. Although no studies have specifically addressed the reasons underlying the low effectiveness of IC-HIVT in Taiwan, research in other regions has highlighted several potential factors. These factors include inadequate adherence to local testing protocols [45], an absence of adequate testing protocols [46], and insufficient awareness and education regarding ICs and the importance of early HIV testing [47, 48]. In this study, we identified many opportunities for early HIV diagnosis through IC-HIVT in Taiwan. We discovered that the prevalence of ICs was significantly higher among PLWH than among matched controls (PLWH vs. matched controls, 42.59% vs. 8.73%). We also discovered intergroup differences in the prevalence of category 1, 2, and 4 ICs from up to 5 years before HIV diagnosis. These findings indicate that implementing IC-HIVT may enable earlier HIV diagnosis by up to 5 years for populations who are at a risk of HIV infection.

Each IC is associated with a unique implementation rate for IC-HIVT. For example, tuberculosis is associated with a high rate of IC-HIVT implementation, whereas ICs such as cervical cancer and hepatitis B or C virus infection are associated with a low rate of IC-HIVT implementation [44]. Understanding the implementation rates for IC-HIVT among different categories of ICs in specific regions may aid in promoting IC-HIVT for these specific categories. To the best of our knowledge, this is the first nationwide survey to examine the differences in HIV and non-HIV prevalence rates between different IC categories as well as the potential for delayed HIV diagnosis. In this study, we analyzed the prevalence of various IC categories among PLWH and the temporal trends associated with potential delays in HIV diagnosis for each IC category. Overall, our findings can be used to formulate tailored strategies to enhance IC-HIVT for each IC category. They can also be used in further studies seeking to investigate the diagnostic delay of HIV infection with various IC categories. According to our previous study, older adults tend to have a higher incidence of delayed HIV diagnosis than younger populations do [49]. Therefore, in this study, we conducted an age-stratified analysis, and we discovered that except for category 1 ICs, older age groups (35 and older) uniformly exhibited a numerically higher number of delayed HIV diagnoses than their younger counterparts (Additional file 5). This finding suggests the need for more targeted interventions aimed at reducing delay in diagnosis among older population.

Our results revealed a slight decrease in HIV diagnostic delay following identification of AOI-related ICs among MSM. Although Taiwan has encouraged self-testing and anonymous HIV testing among MSM, HIV testing based on identification of ICs remains a form of provider-initiated HIV testing. Therefore, the improvements observed in the current study are presumably indicative of growing awareness among health-care professionals regarding the correlation between category 1 ICs and HIV infection resulting in more prompt diagnostic testing. Our study also revealed that the majority of the included patients received their HIV diagnosis within 1.5 months of developing an AOI-related IC. These findings indicate that Taiwanese physicians are generally aware of the correlations between AOIs and HIV. However, the presence of AOI-related ICs indicates advanced HIV infection, which is associated with substantial health-care costs [50] and high rates of morbidity and mortality [10, 51]. Our results revealed an increased risk of all-cause mortality among PLWH with AOI-related ICs. Therefore, emphasis should be placed on strategies that facilitate earlier HIV diagnosis, preferably before the onset of an AOI-related IC. Overall, our data indicate that proactive IC-HIVT targeting category 2 and 3 ICs not only enables enhanced HIV diagnosis before progression to a more severe immunocompromised status associated with category 1 ICs but also reduces HIV diagnosis delays. In this study, 40.4% of PLWH with category 1 ICs developed category 2 ICs beforehand, with a median interval of 58 days (IQR: 0–651 days), whereas 16.4% developed category 3 ICs first, with a median interval of 403 days (IQR: 4–1,130 days). These intervals represent key windows for early intervention, which may substantially hinder progression to severe immunodeficiency, which is characteristic of category 1 ICs.

Because category 2 ICs are common among PLWH (15.68%) and the potential diagnostic delay following a category 2 IC is substantial, enhancing IC-HIVT targeting category 2 ICs is essential. However, implementing this approach for category 2 ICs may be challenging because of the broad spectrum of ICs in this category [18] and the high likelihood of patients with category 2 ICs presenting to non-HIV specialty clinics. Several structural and systemic obstacles may hinder the implementation of IC-HIVT for category 2 ICs. First, the IC-HIVT recommendations outlined in IC management guidelines are inadequate, despite evidence supporting the cost-effectiveness of IC-HIVT in these scenarios [52, 53]. Second, IC-HIVT is typically limited to a few ICs, such as tuberculosis, sexually transmitted infections, and AOIs [25, 44]. Third, many non-HIV specialists may be unaware of long-standing recommendations for IC-HIVT because of a lack of familiarity with these recommendations or a lack of confidence in their ability to explain the need for HIV testing [28].

Although the offer rates for IC-HIVT are low, the uptake of IC-HIVT tends to approach 100% when HIV testing is recommended [27]. This finding indicates that the key barrier to HIV testing is the offering of IC-HIVT rather than test refusal. Therefore, including IC-HIVT in IC guidelines and promoting awareness of these recommendations among physicians of various medical specialties are essential.

Computerized provider order entry can facilitate HIV testing for patients with category 2 ICs and can be implemented with the help of electronic health reminders [26]. Although this approach may not necessarily address all barriers to testing, such as the fear of the negative consequences associated with a positive HIV result (e.g., stigmatization or discrimination), it can aid in overcoming barriers to HIV testing related to category 2 ICs, such as a lack of knowledge regarding category 2 ICs among clinicians. Therefore, governments should establish educational and training programs to increase the awareness of health-care professionals regarding IC-HIVT in the context of IC management [20]. They should also implement policies aimed at simplifying the process of HIV testing by, for example, incorporating opt-out or routine IC-HIVT into care bundles and facilitating access to HIV services while ensuring patient privacy and confidentiality.

Our findings revealed herpes zoster and seborrheic dermatitis as predominant missed diagnostic opportunities for early diagnosis of HIV infection among patients with category 2 ICs (herpes zoster: 5.84%, seborrheic dermatitis: 2.45%). This finding has major implications for general practitioners. Herpes zoster and seborrheic dermatitis are common skin diseases that can be treated at local clinics. General practitioners typically serve as gatekeepers for early HIV diagnoses and HIV specialist referrals [36]. However, not all patients presenting with herpes zoster, seborrheic dermatitis, or other category 2 ICs should undergo HIV screening. Therefore, alternative cost-effective strategies must be developed to improve HIV testing in patients with such ICs. These strategies can be developed by jointly considering the epidemiological characteristics of ICs (e.g., PLWH are more likely to develop herpes zoster at a younger age [54]), the atypical manifestations of ICs in patients with impaired cellular immunity (e.g., PLWH may develop severe variants of herpes zoster or seborrheic dermatitis that may be refractory to treatment [55]), and the risk behaviors of patients with HIV infection.

In this study, we identified a correlation between an increased risk of HIV infection and the presence of a category 4 IC (mononucleosis or mononucleosis-like syndrome). This correlation has major individual and public health implications in terms of avoiding late diagnoses and minimizing the risk of onward transmission. Nevertheless, caution should be exercised while interpreting this finding. Acute retroviral syndrome may be misdiagnosed as infectious mononucleosis because of their similar clinical characteristics and laboratory results [56] and because of the common transmission route of HIV and Epstein–Barr virus (i.e., close contact between sexually active individuals) [56]. Further research is required to differentiate between these two conditions to increase the accuracy of diagnosis by general practitioners and promote the development of effective public interventions.

This study has several strengths. To the best of our knowledge, this is the first nationwide cohort study to examine the correlation between the category of IC and the risk of HIV infection. This is also the first study to investigate the changes that occur in potential diagnostic delays following ICs from different categories. Other strengths of this nationwide study include its extended research period (2009–2015) and its population-based design, with an extended, near-complete follow-up, which considerably minimized selection and referral bias. Overall, our findings can guide public health decision-making in Taiwan and Asia–Pacific countries with similar HIV epidemiology and challenges in HIV care [57]. We recommend that public health authorities initiate integrated campaigns to educate health-care providers on the importance of timely IC-HIVT, including offering advanced training, particularly for physicians in fields that are not typically associated with HIV, to improve management of diverse ICs. We also recommend classification of HIV-related ICs into four categories to facilitate the development of individualized interventions for ICs from different categories.

This study has several limitations. First, the data used in this study were retrieved only from the TCDC and NHIRD. Therefore, the prevalence of specific category 2 ICs, such as unexplained leukocytopenia, unexplained fever, lymphocytic meningitis, subcortical dementia, and unexplained chronic diarrhea, could not be reliably identified, which may explain the lower prevalence of such ICs in our study compared with those in other studies (25.8%–60.7%) [36, 58]. Second, we were unable to differentiate between patients who did not undergo IC-HIVT and those who did undergo VCT but did not receive any medical care, presumably because they were unwilling to receive such care (instead of a lack of awareness regarding ICs among physicians). Third, we were unable to analyze repeat IC episodes because we could not differentiate between conditions that were diagnosed twice and conditions that recurred, which may have resulted in an underestimation of the correlations between ICs and HIV risk. Nonetheless, we identified significant and robust correlations between HIV risk and nearly all of the selected ICs. Fourth, our data set included HIV cases only up to 2015; however, no major changes have been made to the recommendations of IC-HIVT in Taiwan since that time. The only major change was made in 2021, when the recommended age range for tuberculosis HIV screening was expanded from 15–49 years to all ages. Given that the proportion of tuberculosis cases has significantly decreased in Taiwan since 2005 [59], the number of HIV cases with tuberculosis is not likely to have a major effect on the rate of HIV testing in the presence of ICs or on the potential delay of HIV diagnoses [60]. Therefore, our research findings remain applicable to the current scenario in Taiwan. Finally, we did not adjust our statistical models to account for potential confounders or effect modifiers, such as sexual orientation and socioeconomic status.

## Conclusions

Although our findings provide evidentiary support for the IC-HIVT recommendations of the European Centre for Disease Prevention and Control, which are focused on promoting early HIV diagnosis, the implementation of IC-HIVT in Taiwan has been suboptimal. Overall, our findings highlight missed opportunities for early HIV diagnosis through IC-HIVT in Taiwan. Proactive IC-HIVT, particularly for individuals with category 2 ICs, may substantially reduce diagnostic delays of HIV infection and prevent progression to severe immunodeficiency. Therefore, enhancing awareness, incorporating IC-HIVT into routine IC management strategies across disciplines, and simplifying the testing process are crucial steps toward optimizing IC-HIVT and preventing HIV transmission and morbidity.

### Supplementary Information

**Additional file 1.** Definition of four baseline comorbidities and 34 selected ICs from NHIRD by ICD-9 codes, eight selected ICs from NDSS, and the categories thereof.

**Additional file 2.** Risk of HIV diagnosis associated with each IC within 5 years before the index date by logistic regression analysis.

**Additional file 3.** Risk of HIV diagnosis associated with each IC category overall and throughout the five consecutive 1-year intervals before the index date by logistic regression analysis.

**Additional file 4.** Presence of category 2 and 3 ICs before category 1 IC in 1,601 PLWH with category 1 ICs.

**Additional file 5.** Age-stratified analysis of change in HIV diagnostic delay following incidence of IC from each IC category among PLWH from 2009 to 2015.

## Data Availability

The TCDC-operated NDSS and the NHIRD are not publicly available, and restrictions apply to the availability of the databases. Only analyzed data that are de-identified are released to the researchers. All data containing relevant information to support the study findings are provided in the manuscript.

## Abbreviations

AIDS: Acquired immunodeficiency syndrome
AOI: Acquired immunodeficiency syndrome -defining opportunistic illness
HIV: Human immunodeficiency virus
IC: Indicator condition
IC-HIVT: IC-guided HIV testing
IQR: Interquartile range
MSM: Men who have sex with men
N/A: Not available
NDSS: Notifiable diseases surveillance system
NHIRD: National health insurance research database
PLWH: People living with HIV
PWID: People who inject drugs
TCDC: Taiwan Centers for Disease Control
VCT: Voluntary HIV counseling and testing
