# Supplementary material for: Enhancing indicator condition–guided HIV testing in Taiwan: a nationwide case–control study from 2009 to 2015
Source: BMC Public Health. 2024 Apr 5;24:967. doi: 10.1186/s12889-024-18499-6 (PMC10998297; doi:10.1186/s12889-024-18499-6)
Supplement: Supplementary file 1 — Additional file 1. Definition of four baseline comorbidities and 34 selected ICs from NHIRD by ICD-9 codes, eight selected ICs from NDSS, and the categories thereof. [file 12889_2024_18499_MOESM1_ESM.docx]

Additional file 1. Definition of four baseline comorbidities and 34 selected ICs from NHIRD by ICD-9 codes, eight selected ICs from NDSS, and the categories thereof.

|  | | | ICD-9 diagnosis codes^†^ | | | NDSS | |  | | Reference | |  |
| --- | --- | --- | --- | --- | --- | --- | --- | --- | --- | --- | --- | --- |
| **Baseline comorbidities** | | | | | | | | | | | |  |
|  | Cerebrovascular disease | | 430-438 | | |  | |  | | [1] | |  |
|  | Chronic respiratory disease | | 490, 491, 492, 493, 494, 495, 496, 500, 501, 502, 503, 504, 505, 5064 | | |  | |  | | [1] | |  |
|  | Diabetes mellitus | | 250 | | |  | |  | | [1] | |  |
|  | Renal disease | | 582, 5830, 5831, 5832, 5833, 5834, 5835, 5836, 5837, 585, 586, 588 | | |  | |  | | [1] | |  |
| **Category 1 ICs** | | | | | | | | | |  | |  |
|  | Neoplasm | | | | | | | | | |  | |
|  |  | AIDS-defining lymphoma | | 2000, 2002, 2005 |  | | |  | | | [2] | |
|  |  | *Kaposi*’s sarcoma | | 176 |  | | | Other_category1_^‡^ | | | [2] | |
|  |  | Cervical cancer | | 180 |  | | |  |  |  | [2, 3] | |
|  | *Bacterial infection* | | | | | | | | | | | |
|  |  | *Mycobacterium* *tuberculosis* complex | | 010-018 | | |  |  | | | [2, 3] | |
|  |  | Salmonella septicaemia, recurrent | | 0031 | | |  |  | | | [2] | |
|  |  | *Mycobacterium*, other species or unidentified species, disseminated or extrapulmunary | | 0318, 0319 | | |  |  | | | [2] | |
|  |  | *Mycobacterium avium* complex or *Mycobacterium kansasii*, disseminated or extrapulmonary | | 0312 | | |  | Other_category1_^‡^ | | | [2] | |
|  | *Viral infection* | | | | | | | | | | | |
|  |  | CMV disease | | 0785 | | |  |  | | | [2-4] | |
|  |  | HSV infection, other than herpetic genital ulcer | | 0540, 0542-0549 | | |  |  | | | [5] | |
|  |  | Progressive multifocal  leukoencephalopathy | | 0463 | | |  | Other_category1_^‡^ | | | [2, 3] | |
|  | Parasitic infections | | | | | | | | | | | |
|  |  | Toxoplasma encephalitis | | 130 | | |  | Other_category1_^‡^ | | | [3, 4] | |
|  |  | Cryptosporidiosis | | 0074 | | |  |  |  |  | [2] | |
|  | Fungal infections | | | | | | | | | | | |
|  |  | Candidiasis (pulmonary or esophageal) | | 1124, 11284 | | |  |  | | | [2] | |
|  |  | *Penicillium marneffei* infection | 1179 | | |  | |  | | [4] | |  |
|  |  | *Pneumocystis jirovecii* pneumonia | 1363 | | |  | |  | | [2-4] | |  |
|  |  | Cryptococcosis, extra-pulmonary | 1175, 3210 | | |  | |  | | [2] | |  |
|  |  | Coccidioidomycosis, disseminated or extrapulmonary | 1141, 1142, 1143, 1144 | | |  | | Other_category1_^‡^ | | [2] | |  |
|  |  | Histoplasmosis, disseminated or extrapulmonary | 115 | | |  | |  |  | [2, 3] | |  |
| **Category 2 ICs** | | | | | | | | | | |  | |
|  | Herpes zoster infection | | 053 | | | |  | |  | | [6] | |
|  | Candidiasis and candidemia | | 1120, 1121, 1122, 1123, 1125, 1129, 11281, 11282, 11283, 11285, 11289 | | | |  | |  | | [5] | |
|  | Seborrheic dermatitis | | 6901 | | | |  | |  | | [5] | |
|  | Mononeuritis | | 354, 355 | | | |  | |  | | [5] | |
|  | Guillain–barre syndrome | | 3570 | | | |  | |  | | [7] | |
|  | Peripheral neuropathy | | 3564, 3568, 3569, 3571, 3572, 3573, 3574, 3576, 3577, 3578, 3579 | | | |  | |  | | [5] | |
|  | Psoriasis | | 6961 | | | |  | |  | | [8] | |
|  | Oral hairy leukoplakia | | 5286 | | | |  | |  | | [5] | |
|  | Thrombocytopenia | | 2873, 2874, 2875 | | | |  | |  | | [5] | |
|  | Body weight loss | | 7832 | | | |  | |  | | [5] | |
|  | Lymphadenopathy | | 7856 | | | |  | |  | | [5] | |
|  | Invasive pneumococcus infection | |  | | | | √ | |  | |  | |
|  | Primary lung cancer | | 1622, 1623, 1624, 1625, 1628, 1629, 20921, 2312, V1011 | | | |  | | Other_category2_^‡^ | | [2] | |
|  | Anal cancer/dysplasia | | 1542,1543, 2305, 2306, 79670, 79671, 79672, 79673, 79674, 79676 | | | |  | |  |  | [2] | |
| **Category 3 ICs** | | | | | | | | | | |  | |
|  | Hepatitis A virus infection | | 0700, 0701 | | | | √ | |  | | [5] | |
|  | Hepatitis B virus infection | | 0702, 0703, 07042, 07052, V0261 | | | | √ | |  | | [2] | |
|  | Hepatitis C virus infection | | 07041, 07044, 07051, 07054, 07070, 07071, V0262 | | | | √ | |  | | [2] | |
|  | Syphilis | |  | | | | √ | |  | |  | |
|  | Gonorrhoea | |  | | | | √ | |  | |  | |
|  | STDs other than syphilis and gonorrhoea | | 099 | | | |  | |  | | [5] | |
|  | Herpetic genital ulcer | | 0541 | | | |  | |  | | [5] | |
|  | *Entamoeba histolytica* infection | |  | | | | √ | |  | |  | |
|  | *Shigella* infection | |  | | | | √ | |  | |  | |
| **Category 4 ICs** | | | | | | | | | | |  | |
|  | Mononucleosis/mononucleosis-like syndrome | | 075 | | | |  | |  | | [5] | |

Footnote:

^†^ Patients with at least one inpatient or three outpatient claims records of comorbidity- or IC-related ICD-9 codes from NHIRD were regarded as having comorbidities or ICs.

^‡^ We combined some ICs recorded in <3 patients in the control or case group into other categories to prevent reidentification [9]. Other_category1_ included toxoplasma encephalitis, cryptosporidiosis, *Kaposi*’s sarcoma, cervical cancer, progressive multifocal leukoencephalopathy, coccidioidomycosis, histoplasmosis, and *Mycobacterium avium* complex or *Mycobacterium kansasii*, disseminated or extrapulmonary; Other_category2_ included primary lung cancer and anal cancer/dysplasia.

Abbreviation: AIDS, acquired immunodeficiency syndrome; CMV, Cytomegalovirus; HSV, herpes simplex virus; IC, indicator condition; ICD, International Classification of Diseases; NDSS, Notifiable Diseases Surveillance System; NHIRD, National Health Insurance Research Database; STD, sexually transmitted disease.

**Reference**

1. Sundararajan V, Henderson T, Perry C, Muggivan A, Quan H, Ghali WA: **New ICD-10 version of the Charlson comorbidity index predicted in-hospital mortality**. *J Clin Epidemiol* 2004, **57**(12):1288-1294.

2. Koroukian SM, Zhou G, Navale SM, Schiltz NK, Kim U, Rose J, Cooper GS, Moore SE, Mintz LJ, Avery AK *et al*: **Excess cancer prevalence in men with HIV: A nationwide analysis of Medicaid data**. *Cancer* 2022, **128**(10):1987-1995.

3. Glasheen WP, Cordier T, Gumpina R, Haugh G, Davis J, Renda A: **Charlson Comorbidity Index: ICD-9 Update and ICD-10 Translation**. *Am Health Drug Benefits* 2019, **12**(4):188-197.

4. Yen YF, Chen M, Jen IA, Chuang PH, Lee CY, Lin SI, Chen YA: **Short- and Long-term Risks of Highly Active Antiretroviral Treatment with Incident Opportunistic Infections among People Living with HIV/AIDS**. *Sci Rep* 2019, **9**(1):3476.

5. **The International Classification of Diseases, 9th Revision, Clinical Modification** [<http://icd9cm.chrisendres.com/index.php>]

6. Weinmann S, Vandermeer M, Roberts M, Mullooly J, Chun C: **Positive Predictive Value of ICD-9 Code for Herpes Zoster Among Children During the Varicella Vaccine Era**. *Pediatr Infect Dis J* 2016, **35**(4):459-460.

7. Shui IM, Rett MD, Weintraub E, Marcy M, Amato AA, Sheikh SI, Ho D, Lee GM, Yih WK: **Guillain-Barré syndrome incidence in a large United States cohort (2000-2009)**. *Neuroepidemiology* 2012, **39**(2):109-115.

8. Asgari MM, Wu JJ, Gelfand JM, Salman C, Curtis JR, Harrold LR, Herrinton LJ: **Validity of diagnostic codes and prevalence of psoriasis and psoriatic arthritis in a managed care population, 1996-2009**. *Pharmacoepidemiol Drug Saf* 2013, **22**(8):842-849.

9. Lin LY, Warren-Gash C, Smeeth L, Chen PC: **Data resource profile: the National Health Insurance Research Database (NHIRD)**. *Epidemiol Health* 2018, **40**:e2018062.
