# Supplementary material for: Enhancing indicator condition–guided HIV testing in Taiwan: a nationwide case–control study from 2009 to 2015
Source: BMC Public Health. 2024 Apr 5;24:967. doi: 10.1186/s12889-024-18499-6 (PMC10998297; doi:10.1186/s12889-024-18499-6)
Supplement: Supplementary file 2 — Additional file 2. Risk of HIV diagnosis associated with each IC within 5 years before the index date by logistic regression analysis. [file 12889_2024_18499_MOESM2_ESM.docx]

Additional file 2. Risk of HIV diagnosis associated with each IC within 5 years before the index date by logistic regression analysis.

|  | | All  (N=157,817) | Control group (N=143,470) | Case group  (N=14,347) | *P*-value | Crude ORs (95% CI) | Adjusted ORs (95% CI)^‡^ |
| --- | --- | --- | --- | --- | --- | --- | --- |
| All ICs | | 18,634  (11.81) | 12,523  (8.73) | 6,111  (42.59) | <0.001 | 7.76 (7.47–8.06)^***^ | 8.76 (8.42–9.11)^***^ |
| Category 1 ICs | | 3,103  (1.97) | 1,502  (1.05) | 1,601  (11.16) | <0.001 | 11.87 (11.04–12.77)^***^ | 12.74 (11.83–13.72)^***^ |
|  | *Mycobacterium* *tuberculosis* complex | 651  (0.41) | 394  (0.27) | 257  (1.79) | <0.001 | 6.62 (5.66–7.76)^***^ | 7.15 (6.09–8.40)^***^ |
|  | *Penicillium marneffei* infection | 224  (0.14) | 167  (0.12) | 57  (0.40) | <0.001 | 3.43 (2.54–4.63)^***^ | 3.53 (2.61–4.78)^***^ |
|  | CMV disease | 266  (0.17) | 20  (0.01) | 246  (1.71) | <0.001 | 124.86 (79.17–196.93)^***^ | 131.66 (83.25–208.21)^***^ |
|  | Candidiasis (pulmonary or esophageal) | 143  (0.09) | 8  (0.01) | 135  (0.94) | <0.001 | 170.22 (83.42–347.33)^***^ | 179.13 (87.61–366.28)^***^ |
|  | AIDS-defining lymphoma | 38  (0.02) | 22  (0.02) | 16  (0.11) | <0.001 | 7.28 (3.82–13.87)^***^ | 7.37 (3.86–14.07)^***^ |
|  | *Pneumocystis jirovecii* pneumonia | 850  (0.54) | 6  (0.004) | 844  (5.88) | <0.001 | 999.99 (669.38–999.99)^***^ | 999.99 (698.14–999.99)^***^ |
|  | Cryptococcosis, extra-pulmonary | 106  (0.07) | 14  (0.01) | 92  (0.64) | <0.001 | 65.85 (37.55–115.48)^***^ | 70.06 (39.78–123.39)^***^ |
|  | *Salmonella* septicaemia, recurrent | 83  (0.05) | 6  (0.004) | 77  (0.54) | <0.001 | 128.75 (56.13–295.30)^***^ | 145.82 (63.31–335.83)^***^ |
|  | *Mycobacterium*, other species or unidentified species, disseminated or extrapulmunary | 44  (0.03) | 12  (0.01) | 32  (0.22) | <0.001 | 26.70 (13.75–51.83)^***^ | 28.41 (14.55–55.45)^***^ |
|  | HSV infection, other than herpetic genital ulcer | 1,144  (0.72) | 868  (0.61) | 276  (1.92) | <0.001 | 3.23 (2.82–3.70) ^***^ | 3.23 (2.82–3.71)^***^ |
|  | Other_category1_ | 94  (0.06) | 20  (0.01) | 74^†^  (0.52) | <0.001 | 37.04 (22.61–60.68)^***^ | 37.19 (22.63–61.13)^***^ |
| Category 2 ICs | | 8,744  (5.54) | 6,494  (4.53) | 2,250  (15.68) | <0.001 | 3.92 (3.73–4.13)^***^ | 4.19 (3.98–4.42)^***^ |
|  | Herpes zoster infection | 2,054  (1.30) | 1,216  (0.85) | 838  (5.84) | <0.001 | 7.26 (6.64–7.94)^***^ | 7.57 (6.91–8.29)^***^ |
|  | Candidiasis and candidemia | 1,319  (0.84) | 617  (0.43) | 702  (4.89) | <0.001 | 11.91 (10.67–13.29)^***^ | 13.25 (11.83–14.84)^***^ |
|  | Seborrheic dermatitis | 2,317  (1.47) | 1,965  (1.37) | 352  (2.45) | <0.001 | 1.81 (1.62–2.03)^***^ | 1.82 (1.63–2.05)^***^ |
|  | Mononeuritis | 1,171  (0.74) | 1,084  (0.76) | 87  (0.61) | 0.047 | 0.80 (0.64–0.99)^*^ | 0.82 (0.66–1.03) |
|  | Guillain–barre syndrome | 24  (0.02) | 17  (0.01) | 7  (0.05) | <0.001 | 4.12 (1.71–9.94)^***^ | 4.10 (1.70–9.90)^**^ |
|  | Peripheral neuropathy | 473  (0.30) | 429  (0.30) | 44  (0.31) | 0.873 | 1.03 (0.75–1.40) | 1.26 (0.92–1.73) |
|  | Psoriasis | 797  (0.51) | 696  (0.49) | 101  (0.70) | <0.001 | 1.46 (1.18–1.80)^***^ | 1.48 (1.20–1.83)^***^ |
|  | Oral hairy leukoplakia | 164  (0.10) | 146  (0.10) | 18  (0.13) | 0.401 | 1.23 (0.76–2.01) | 1.28 (0.78–2.10) |
|  | Thrombocytopenia | 447  (0.28) | 226  (0.16) | 221  (1.54) | <0.001 | 9.90 (8.22–11.93) ^***^ | 10.60 (8.78–12.79)^***^ |
|  | Body weight loss | 284  (0.18) | 188  (0.13) | 96  (0.67) | <0.001 | 5.15 (4.02–6.58)^***^ | 5.60 (4.36–7.19)^***^ |
|  | Lymphadenopathy | 413  (0.26) | 241  (0.17) | 172  (1.20) | <0.001 | 7.21 (5.93–8.78)^***^ | 7.25 (5.95–8.83)^***^ |
|  | Invasive pneumococcus infection | 14  (0.01) | 9  (0.01) | 5  (0.03) | <0.001 | 5.59 (1.88–16.68)^**^ | 6.14 (2.04–18.51)^**^ |
|  | Other_category2_ | 85  (0.05) | 75  (0.05) | 10  (0.07) | 0.391 | 1.33 (0.69–2.58) | 1.32 (0.68–2.55) |
| Category 3 ICs | | 9,098  (5.76) | 5,299  (3.69) | 3,799  (26.48) | <0.001 | 9.39 (8.97–9.84)^***^ | 10.22 (9.74–10.72)^***^ |
|  | Hepatitis A virus infection | 12  (0.01) | 9  (0.01) | 3  (0.02) | 0.055 | 3.34 (0.90–12.33) | 3.15 (0.85–11.63) |
|  | Hepatitis B virus infection | 4,599  (2.91) | 4,161  (2.90) | 438  (3.05) | 0.300 | 1.06 (0.96–1.17) | 1.07 (0.97–1.18) |
|  | Hepatitis C virus infection | 969  (0.61) | 788  (0.55) | 181  (1.26) | <0.001 | 2.31 (1.97–2.72)^***^ | 2.48 (2.10–2.92)^***^ |
|  | Syphilis | 2,958  (1.87) | 125  (0.09) | 2,833  (19.75) | <0.001 | 282.16 (235.65–337.85)^***^ | 286.29 (239.06–342.86)^***^ |
|  | Gonorrhoea | 645  (0.41) | 178  (0.12) | 467  (3.26) | <0.001 | 27.06 (22.75–32.18)^***^ | 26.84 (22.56–31.93)^***^ |
|  | STDs other than syphilis and gonorrhoea | 327  (0.21) | 124  (0.09) | 203  (1.41) | <0.001 | 16.59 (13.26–20.76) | 16.61 (13.27–20.79) |
|  | Herpetic genital ulcer | 136 (0.09) | 87  (0.06) | 49  (0.34) | <0.001 | 5.65 (3.98–8.02)^***^ | 5.53 (3.89–7.86)^***^ |
|  | Entamoeba histolytica infection | 82  (0.05) | 6  (0.004) | 76  (0.53) | <0.001 | 127.06 (55.38–291.56)^***^ | 125.90 (54.77–289.38)^***^ |
|  | Shigella infection | 11  (0.01) | 7  (0.005) | 4  (0.03) | 0.002 | 5.78 (1.69–19.70)^**^ | 5.63 (1.65–19.23)^**^ |
| Category 4 IC | | 170  (0.11) | 31  (0.02) | 139  (0.97) | <0.001 | 45.27 (30.66–66.84)^***^ | 46.49 (31.45–68.72)^***^ |

Footnote:

^†^15 in Disseminated *Mycobacterium avium* complex infection (15/14347, 0.10%), 18 in toxoplasma encephalitis (18/14,347, 0.13%), 28 in *Kaposi*’s sarcoma (28/14,347, 0.20%).

^‡^ Baseline cerebral vascular disease, chronic obstructive pulmonary disorder, diabetes mellitus, renal disease, age group, and gender were incorporated into the binary logistic regression.

^*^*P*-value<0.05; ^**^ *P*-value<0.01; ^***^*P*-value<0.001.

Abbreviation:

AIDS, acquired immunodeficiency syndrome; CI, confidence interval; CMV, Cytomegalovirus; HSV, herpes simplex virus; IC, indicator condition; OR, odds ratio; STD, sexually transmitted disease.
