# Supplementary material for: Enhancing indicator condition–guided HIV testing in Taiwan: a nationwide case–control study from 2009 to 2015
Source: BMC Public Health. 2024 Apr 5;24:967. doi: 10.1186/s12889-024-18499-6 (PMC10998297; doi:10.1186/s12889-024-18499-6)
Supplement: Supplementary file 3 — Additional file 3. Risk of HIV diagnosis associated with each IC category overall and throughout the five consecutive 1-year intervals before the index date by logistic regression analysis. [file 12889_2024_18499_MOESM3_ESM.docx]

Additional file 3. Risk of HIV diagnosis associated with each IC category overall and throughout the five consecutive 1-year intervals before the index date by logistic regression analysis.

|  | | | | All  (N=157,817) | Control group^†^ (N=143,470) | Case group^†^  (N=14,347) | Crude ORs (95% CI) | Adjusted ORs (95% CI)^‡^ |
| --- | --- | --- | --- | --- | --- | --- | --- | --- |
| Any IC categories | | | | 18,634  (11.81) | 12,523  (8.73) | 6,111  (42.59) | 7.76 (7.47–8.06)^***^ | 8.76 (8.42–9.11)^***^ |
|  | Between 0 and 1^st^ year | | | 4,969  (3.14) | 1,637  (1.14) | 3,332  (23.22) | 26.21 (24.62–27.89)^***^ | 27.25 (25.58–29.02)^***^ |
|  | Between 1^st^ and 2^nd^ year | | | 2,645  (1.68) | 1,861  (1.30) | 784  (5.46) | 4.40 (4.04–4.79) | 4.52 (4.15–4.92)^***^ |
|  | Between 2^nd^ and 3^rd^ year | | | 2,711  (1.71) | 2,016  (1.41) | 695  (4.84) | 3.57 (3.27–3.90)^***^ | 3.66 (3.35–4.00)^***^ |
|  | Between 3^rd^ and 4^th^ year | | | 3,063  (1.94) | 2,429  (1.69) | 634  (4.42) | 2.69 (2.46–2.94)^***^ | 2.75 (2.51–3.00)^***^ |
|  | Between 4^th^ and 5^th^ year | | | 5,323  (3.37) | 4,600  (3.21) | 723  (5.04) | 1.60 (1.48–1.74)^***^ | 1.65 (1.52–1.79) |
| Category 1 ICs | | | | 3,103  (1.97) | 1,502  (1.05) | 1,601  (11.16) | 11.87 (11.04–12.77)^***^ | 12.74 (11.83–13.72)^***^ |
|  | | | Between 0 and 1^st^ year | 1,544  (0.98) | 204  (0.14) | 1,340  (9.34) | 72.35 (62.37–83.92)^***^ | 77.85 (67.00–90.47) |
|  |  |  | Between 1^st^ and 2^nd^ year | 293  (0.19) | 229  (0.16) | 64  (0.45) | 2.81 (2.13–3.70)^***^ | 2.93 (2.22–3.87)^***^ |
|  |  |  | Between 2^nd^ and 3^rd^ year | 293  (0.19) | 241  (0.17) | 52  (0.36) | 2.16 (1.60–2.92)^***^ | 2.16 (1.60–2.92)^***^ |
|  |  |  | Between 3^rd^ and 4^th^ year | 373  (0.24) | 308  (0.21) | 65  (0.45) | 2.12 (1.62–2.77)^***^ | 2.15 (1.65–2.82)^***^ |
|  |  |  | Between 4^th^ and 5^th^ year | 600  (0.38) | 520  (0.36) | 80  (0.56) | 1.55 (1.22–1.96)^***^ | 1.57 (1.24–1.99)^***^ |
| Category 2 ICs | | | | 8,744  (5.54) | 6,494  (4.53) | 2,250  (15.68) | 3.92 (3.73–4.13)^***^ | 4.19 (3.98–4.42)^***^ |
|  | | Between 0 and 1^st^ year | | 2,233  (1.41) | 1,049  (0.73) | 1,184  (8.25) | 12.21 (11.22–13.29)^***^ | 12.80 (11.74–13.95)^***^ |
|  |  | Between 1^st^ and 2^nd^ year | | 1,492  (0.95) | 1,156  (0.81) | 336  (2.34) | 2.96 (2.61–3.34)^***^ | 3.02 (2.67–3.42)^***^ |
|  |  | Between 2^nd^ and 3^rd^ year | | 1,430  (0.91) | 1,165  (0.81) | 265  (1.85) | 2.30 (2.01–2.63)^***^ | 2.37 (2.07–2.72)^***^ |
|  |  | Between 3^rd^ and 4^th^ year | | 1,472  (0.93) | 1,262  (0.88) | 210  (1.46) | 1.67 (1.45–1.94)^***^ | 1.70 (1.47–1.97)^***^ |
|  |  | Between 4^th^ and 5^th^ year | | 2,117  (1.34) | 1,862  (1.30) | 255  (1.78) | 1.38 (1.21–1.57)^***^ | 1.43 (1.25–1.63)^***^ |
| Category 3 ICs | | | | 9,098  (5.76) | 5,299  (3.69) | 3,799  (26.48) | 9.39 (8.97–9.84)^***^ | 10.22 (9.74–10.72)^***^ |
|  | Between 0 and 1^st^ year | | | 2,640  (1.67) | 555  (0.39) | 2,085  (14.53) | 43.79 (39.80–48.17)^***^ | 44.57 (40.50–49.06)^***^ |
|  | Between 1^st^ and 2^nd^ year | | | 1,115  (0.71) | 645  (0.45) | 470  (3.28) | 7.50 (6.65–8.46)^***^ | 7.74 (6.86–8.73)^***^ |
|  | Between 2^nd^ and 3^rd^ year | | | 1,207  (0.76) | 771  (0.54) | 436  (3.04) | 5.80 (5.15–6.53)^***^ | 5.93 (5.27–6.69)^***^ |
|  | Between 3^rd^ and 4^th^ year | | | 1,384  (0.88) | 987  (0.69) | 397  (2.77) | 4.11 (3.65–4.63)^***^ | 4.21 (3.74–4.74)^***^ |
|  | Between 4^th^ and 5^th^ year | | | 2,752  () | 2,341  (1.63) | 411  (2.86) | 1.78 (1.60–1.98)^***^ | 1.81 (1.63–2.02)^***^ |
| Category 4 IC | | | | 170  (1.74) | 31  (0.02) | 139  (0.97) | 45.27 (30.66–66.84)^***^ | 46.49 (31.45–68.72)^***^ |
|  | | | Between 0 and 1^st^ year | 105  (0.07) | 3  (0.002) | 102  (0.71) | 341.09 (108.42–999.99)^***^ | 349.22 (111.00–999.99)^***^ |
|  |  |  | Between 1^st^ and 2^nd^ year | 16  (0.01) | 5  (0.003) | 11  (0.08) | 21.97 (7.64–63.20)^***^ | 22.43 (7.78–64.65)^***^ |
|  |  |  | Between 2^nd^ and 3^rd^ year | 20  (0.13) | 8  (0.01) | 12  (0.08) | 15.00 (6.13–36.69)^***^ | 14.67 (6.00–35.90)^***^ |
|  |  |  | Between 3^rd^ and 4^th^ year | 13  (0.008) | 5  (0.003) | 8  (0.06) | 16.01 (5.24–48.94) | 16.61 (5.41–50.98)^***^ |
|  |  |  | Between 4^th^ and 5^th^ year | 16  (0.01) | 10  (0.01) | 6  (0.04) | 6.00 (2.18–16.52)^***^ | 6.31 (2.28–17.48)^***^ |

Footnote:

^†^ All the comparisons of each IC category in each 1-year interval between case and control groups were *P*<0.001.

^‡^ Baseline cerebral vascular disease, chronic obstructive pulmonary disorder, diabetes mellitus, renal disease, age group, and gender were incorporated into the binary logistic regression

^*^*P*-value<0.05; ^**^ *P*-value<0.01; ^***^*P*-value<0.001

Abbreviation:

CI, confidence interval; OR, odds ratio.
