# Supplementary material for: Enhancing indicator condition–guided HIV testing in Taiwan: a nationwide case–control study from 2009 to 2015
Source: BMC Public Health. 2024 Apr 5;24:967. doi: 10.1186/s12889-024-18499-6 (PMC10998297; doi:10.1186/s12889-024-18499-6)
Supplement: Supplementary file 4 — Additional file 4. Presence of category 2 and 3 ICs before category 1 IC in 1,601 PLWH with category 1 ICs. [file 12889_2024_18499_MOESM4_ESM.docx]

Additional file 4. Presence of category 2 and 3 ICs before category 1 IC in 1,601 PLWH with category 1 ICs.

|  | 1,601 PLWH with category 1 ICs, n (%) | Median days between category 2 or 3 ICs and category 1 ICs (IQR) |
| --- | --- | --- |
| Category 2 before category 1 | 641 (40.4) | 58 (0-651) |
| Category 3 before category 1 | 262 (16.4) | 403 (4-1,130) |

Abbreviation: IC, indicator condition; IQR, interquartile range; PLWH, people living with HIV.
