# Supplementary material for: Enhancing indicator condition–guided HIV testing in Taiwan: a nationwide case–control study from 2009 to 2015
Source: BMC Public Health. 2024 Apr 5;24:967. doi: 10.1186/s12889-024-18499-6 (PMC10998297; doi:10.1186/s12889-024-18499-6)
Supplement: Supplementary file 5 — Additional file 5. Age-stratified analysis of change in HIV diagnostic delay following incidence of IC from each IC category among PLWH from 2009 to 2015. [file 12889_2024_18499_MOESM5_ESM.docx]

Additional file 5. Age-stratified analysis of change in HIV diagnostic delay following incidence of IC from each IC category among PLWH from 2009 to 2015.

|  | | | | | | Overall | 2009 | 2010 | 2011 | 2012 | 2013 | | 2014 | 2015 | *P*-value |
| --- | --- | --- | --- | --- | --- | --- | --- | --- | --- | --- | --- | --- | --- | --- | --- |
| All routes of HIV transmission, median day (IQR) | | | | | | | | | | | | | | | |
|  | Category 1 IC | | | | |  |  |  |  |  |  |  | |  |  |
|  |  | 15≦~<25 | | | | 16.5  (8–178.5) | 17  (8–5.25) | 24  (12–103) | 12  (3–357) | 12  (8–103) | 20  (12–95) | 17.5  (10–105.5) | | 15  (8–212) | 0.821 |
|  |  | 25≦~<35 | | | | 14  (7–33.5) | 17  (7-46) | 17.5  (7.5–26.5) | 11  (5–19) | 15  (6–217) | 15.5  (7–48) | 12  (7–18) | | 14  (7–43) | 0.276 |
|  |  | 35≦ | | | | 16  (8–40) | 15  (8–135) | 17  (8–47.5) | 16  (6–29) | 17  (8–76) | 16  (7–13.5) | 15  (7–28) | | 14  (8–29) | 0.890 |
|  | Category 2 IC | | | | |  |  |  |  |  |  |  | |  |  |
|  |  | 15≦~<25 | | | | 191  (40–622) | 109  (24–695.5) | 185  (47–579) | 97  (32–324) | 216  (64–604.5) | 328  (40–699) | 323  (44–799) | | 179  (39–776) | 0.244 |
|  |  | 25≦~<35 | | | | 257  (24–903) | 98.5  (21–621) | 328  (44–904) | 138  (16–747.5) | 267  (28–1032) | 244.5  (25–738.5) | 352  (23–941) | | 294.5  (22–985.5) | 0.155 |
|  |  | 35≦ | | | | 441  (60–1089) | 468  (49–1067) | 579  (49–1158) | 292  (33–951) | 390.5  (63.5–1060.5) | 444  (81–1001) | 473  (64–990) | | 393  (81–1216) | 0.601 |
|  | Category 3 IC | | | | |  |  |  |  |  |  |  | |  |  |
|  |  | | 15≦~<25 | | | 190  (14–705) | 273  (20–936) | 130.5  (9–817) | 152  (13.5–599) | 266.5  (16–741) | 215  (17–606) | 176  (14–704) | | 176  (14–704) | 0.586 |
|  |  |  | 25≦~<35 | | | 241  (13–1039) | 447  (14–1207) | 350  (13–1090) | 313.5  (17–1062) | 206  (12–927) | 215  (13–950) | 306  (13.5–1126.5) | | 72  (10–898) | 0.016 |
|  |  |  | 35≦ | | | 281  (13–1168) | 112  (11–976) | 281  (13–1270) | 124  (11–940) | 354  (12–1171) | 264  (13.5–1134.5) | 437  (12–1177) | | 414  (14–1388) | 0.414 |
|  | Category 4 IC | | | | |  |  |  |  |  |  |  | |  |  |
|  |  | | | 15≦~<25 | | 74  (27–303) | 72  (33–1041) | 188  (32–623) | 147  (14–357) | 75.5  (28–171) | 37  (18–140) | 67  (6–172) | | 67  (6–172) | 0.534 |
|  |  |  |  | 25≦~<35 | | 70.5  (34–579) | 90  (16–103) | 78.5  (69–88) | 64  (40.5–212) | 217  (39–876) | 55.5  (28–148) | 222.5  (40.5–957.5) | | 313  (34–1322) | 0.870 |
|  |  |  |  | 35≦ | | 150  (36–1011) | 301.5  (36–567) | 227  (165–1028) | 1165  (–-1767) | 87.5  (43–123.5) |  | 357.5  (67–648) | | 511  (11–1011) | 0.756 |
|  | All ICs | | | | |  |  |  |  |  |  |  | |  |  |
|  |  | | | | 15≦~<25 | 191  (17–704) | 242  (21.5–936) | 149  (15–774) | 129.5  (14–596) | 252.5  (21.5–751.5) | 208  (20–667) | 167  (14–789) | | 197.5  (17–704) | 0.316 |
|  |  |  |  |  | 25≦~<35 | 248.5  (15–1007) | 376  (18–1077) | 334  (18–1027) | 218.5  (16–976) | 246  (13–1009) | 238  (16–986) | 301  (16–1088) | | 159  (13–947) | 0.385 |
|  |  |  |  |  | 35≦ | 360  (18–1170) | 308  (17–1114) | 417.5  (18–1267) | 198  (15–972) | 367  (19–1171) | 369  (20–1136) | 482  (19–1141) | | 395  (16–1335) | 0.323 |

Note: In the original study, we divided the ages of the subjects into five groups. However, due to NHIRD regulations that do not permit the exportation of patient counts less than three [40], many counts could not be exported under the five-group categorization. Therefore, in the age stratification analysis, we combined the age groups '35≦~<45', '45≦~<55', and '>55' into a single category '≧35 '. Empty cells signify that there are 'fewer than 3 patients'."

Abbreviations: IC, indicator condition; IQR, interquartile range; PLWH, people living with HIV.
